# The Suzuki Reaction in Aqueous Media Promoted by P, N Ligands

**DOI:** 10.3390/molecules16086215

**Published:** 2011-07-25

**Authors:** Jason A. Weeden, Rongcai Huang, Kathryn D. Galloway, Phillip W. Gingrich, Brian J. Frost

**Affiliations:** Department of Chemistry, University of Nevada, Reno NV 89557, USA

**Keywords:** C–C coupling, aqueous phase Suzuki reaction, 1,3,5-triaza-7-phosphaadamantante (PTA), water-soluble phosphine ligands

## Abstract

The synthesis and structure of palladium complexes of trisubstituted PTA derivatives, PTA_R3_, are described. Water-soluble phosphine ligands 1,3,5-triaza-7-phosphaadmantane (PTA), tris(aminomethyl)phosphine trihydrobromide, tri(aminomethyl) phosphine, 3,7-dimethyl-1,5,7-triaza-3-phosphabicyclo[3,3,1]nonane (RO-PTA), 3,7-diacetyl-1,3,7-triaza-5-phosphabicyclo[3.3.1]nonane (DAPTA), lithium 1,3,5-triaza-7-phosphaadamantane-6-carboxylate (PTA-CO_2_Li), 2,4,6-triphenyl-1,3,5-triaza-7-phosphatricyclo[3.3.1.1]decane, and 2,4,6-triphenyl-1,3,5-triaza-7-phosphatricyclo[3.3.1.1]decane were used as ligands for palladium catalyzed Suzuki reactions in aqueous media. RO-PTA in combination with palladium acetate or palladium chloride was the most active catalyst for Suzuki cross coupling of aryl bromides and phenylboronic acid at 80 °C in 1:1 water:acetonitrile. The activity of Pd(II) complexes of RO-PTA is comparable to PPh_2_(*m*-C_6_H_4_SO_3_Na) (TPPMS) and P(*m*-C_6_H_4_SO_3_Na)_3_ (TPPTS) and less active than tri(4,6-dimethyl-3-sulfonatophenyl)phosphine trisodium salt (TXPTS). Activated, deactivated, and sterically hindered aryl bromides were examined, with yields ranging from 50% to 90% in 6 h with 5% palladium precatalyst loading. X-ray crystal structures of (RO-PTA)PdCl_2_, (PTA_R3_)_2_PdCl_2_ (R = Ph, *p-tert-*butylC_6_H_5_), and PTA_R3_ (R = *p*-*tert*-butylC_6_H_5_) are reported.

## 1. Introduction

Transition metal catalyzed C–C bond formations are one of the most important transformations in organic synthesis [1]. Pd-catalyzed coupling reactions are among the most efficient methods to construct carbon-carbon and carbon-heteroatom bonds [2,3,4,5,6,7,8,9,10]. Heck [2,3], Suzuki [2,4], Stille [5,6], Sonogashira [7,8], and Buchwald-Hartwig [9,10] couplings are widely used to synthesize natural products, materials, and polymers. Since Casalnuovo’s initial report of palladium-catalyzed cross-coupling reactions in aqueous solvents using TPPMS/Pd(OAc)_2_ [11], a variety of catalytic systems in aqueous media have been developed and used for cross-coupling reactions [12,13,14,15,16,17,18,19,20,21,22,23,24,25,26,27]. Some recent examples of aqueous phase Suzuki reactions involve sterically demanding ligands [12,22,23], ligand free palladium catalysts [14,24], palladium nanoparticles on ionic liquids co-polymerized with styrene [15], and ZrO_2_ impregnated with palladium nanoparticles [17].

**Figure 1:**
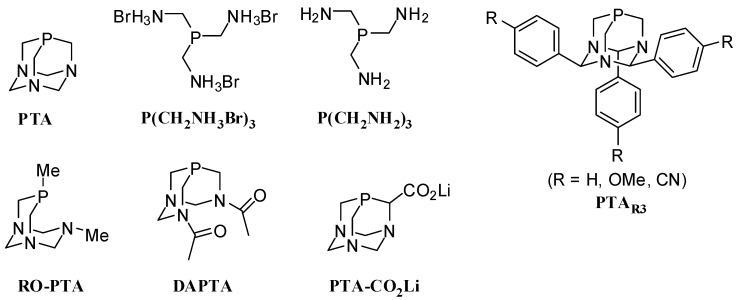
PTA and derivatives of PTA.

We have been interested in the chemistry of the neutral, air-stable and water soluble 1,3,5-triaza-7-phosphaadmantane (PTA) [28,29,30,31,32,33,34]. Several PTA derivatives have been published in the literature including ring-opened PTA (RO-PTA) [35,36], DAPTA [28,36,37], PTA-CO_2_Li [33], P(CH_2_NH_3_Br)_3_ [38], P(CH_2_NH_2_)_3_ [30], and PTA_R3_ (R = Ph, *p*-PhOCH_3_, *p*-PhCN) [30] (Figure 1). Due to the excellent donating properties and water solubility of PTA Joó, Darensbourg, and others introduced PTA to aqueous phase catalysis [39,40,41,42,43,44,45]. PTA and complexes of PTA have been utilized as catalysts for other reactions including but not limited to aqueous or aqueous biphasic hydrogenation of alkenes and aldehydes [41,42,43], hydroamination [46], the Baylis-Hillman reaction [47,48], Sonagashira coupling [49], Huisgen cycloadditions of azides and terminal alkynes [50], and allylation of acetylacetone by allylic carbonates [51]. PTA has also been used as a ligand in regioselective Suzuki reactions of dihaloimidazoles and dihalooxazoles [52].

## 2. Results and Discussion

**Scheme 1:**
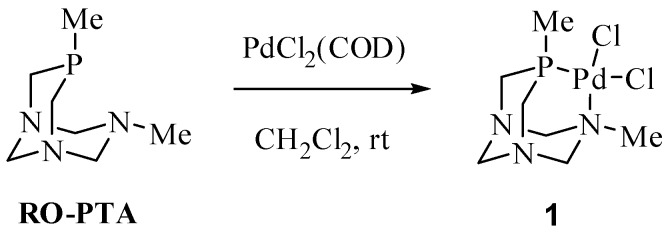
Synthesis of (RO-PTA)PdCl_2_ (**1**).

**Figure 2:**
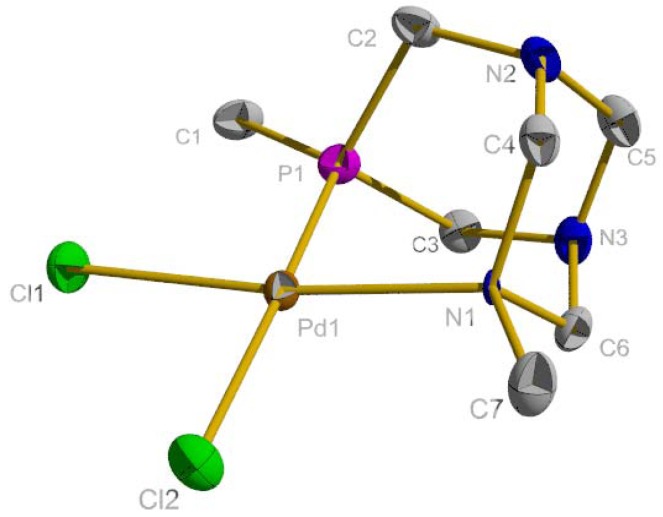
Thermal ellipsoid plot (50% probability) of **1**. Hydrogen atoms have been omitted for clarity. Selected bond lengths (Å) and angles (deg.): Pd1-N1 = 2.131(3), Pd1-P1 = 2.1884(12), Pd1-Cl1 = 2.3007(11), Pd1-Cl2 = 2.4074(11), N1-Pd1-P1 = 85.42(8), N1-Pd1-Cl1 = 173.12(9), P1-Pd1-Cl2 = 177.82(4), P1-Pd1-Cl1 = 89.94(4).

Palladium complex **1** was obtained as a yellow solid in 78% yield by reacting RO-PTA and PdCl_2_(COD) in methylene chloride at room temperature for one hour (Scheme 1). Compound **1** is slightly soluble in organic solvents and soluble in water; however, aqueous solutions of **1** form black precipitates over several hours indicating decomposition. The square planar palladium center of **1**, Figure 2, contains similar bond lengths and bond angles as the analogous palladium acetate derivative synthesized by Peruzzini and coworkers [53]. The ^31^P-NMR spectrum of **1** contains a singlet at −26.6 ppm in D_2_O, downfield from the *cis*-palladium acetate derivative (−46.6 ppm) in CD_2_Cl_2_ [53]. Peruzzini and coworkers obtained the ^31^P-NMR spectrum of *trans*-PdCl_2_{κ^1^-P-(RO-PTA)}_2_ which exhibits a singlet at −49.0 ppm in CD_2_Cl_2_ and −31.2 ppm in D_2_O showing that the NMR resonances can shift significantly downfield upon changing the solvent from CD_2_Cl_2_ to D_2_O [53].

**Scheme 2:**
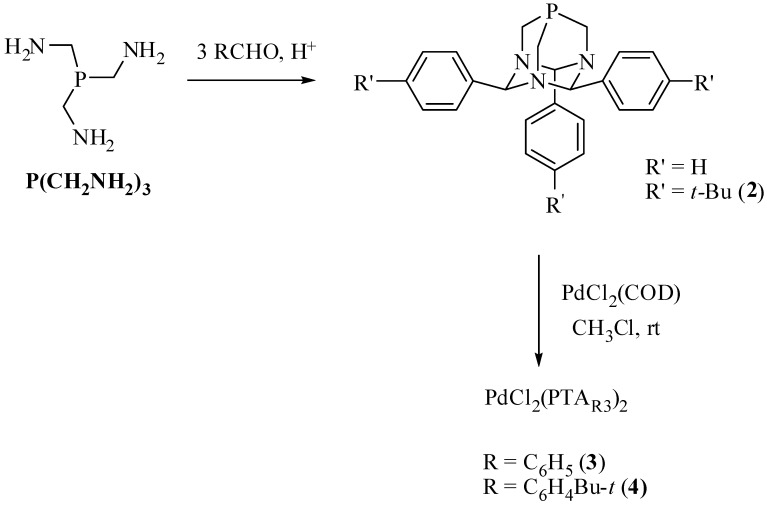
Synthesis of PTA_R3_ and palladium complexes.

**Figure 3:**
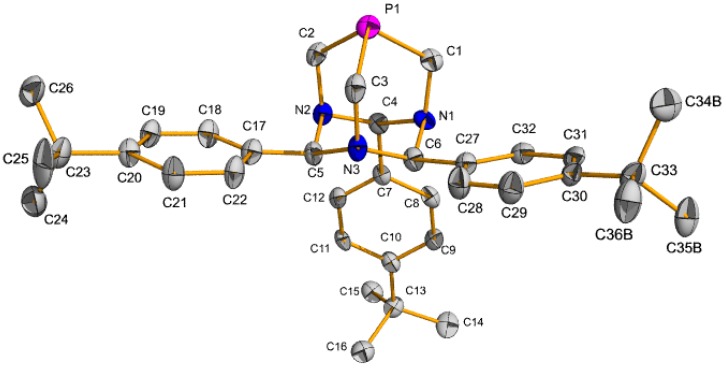
Thermal ellipsoid plot (50% probability) of **2**. Hydrogen atoms have been omitted for clarity. Selected bond lengths (Å) and angles (deg.): P1-C_ave_ = 1.858(4), N-C_ave_ = 1.481(4), C4-C7 = 1.516(5), C5-C17 = 1.520(5), C6-C27 = 1.515(5), C-P1-C_ave_ = 95.65(18).

**Figure 4:**
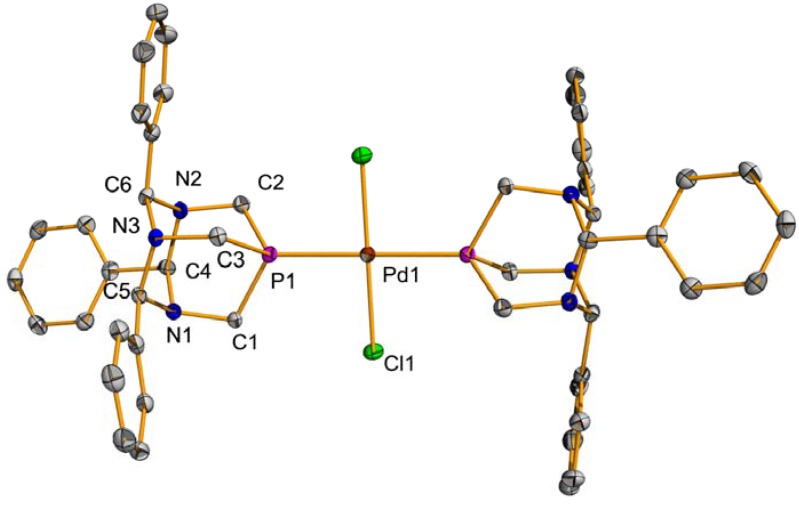
Thermal ellipsoid plot (50% probability) of **3**. Hydrogen atoms have been omitted for clarity. Selected bond lengths (Å) and angles (deg.): Pd1-Cl1 = 2.2930(7), Pd1-P1 = 2.2895(7), P-Pd-P1 = 180.00(6), Cl-Pd1-Cl1 = 180.00(5), Cl1-Pd1-P1 = 87.78(3).

**Figure 5:**
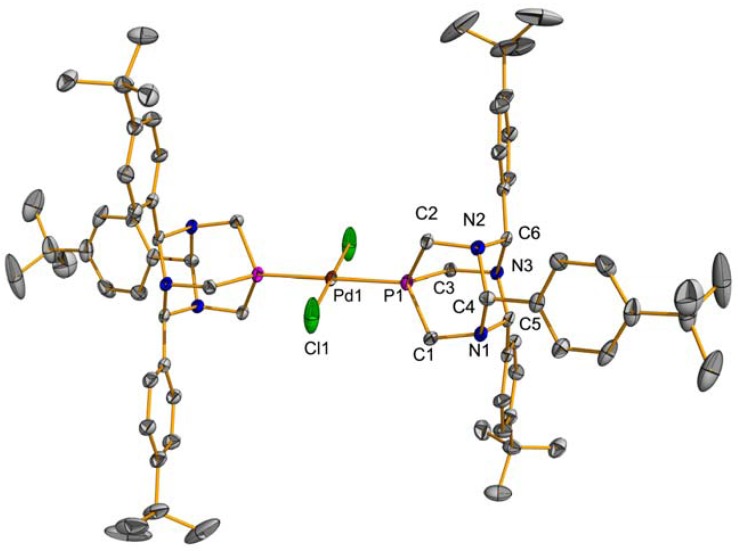
Thermal ellipsoid plot (50% probability) of **4**. Hydrogen atoms have been omitted for clarity. Selected bond lengths (Å) and angles (deg.): Pd1-Cl1 = 2.2911(17), Pd1-P1 = 2.2959(13), P-Pd-P1 = 180.0, Cl-Pd1-Cl1 = 180.0, Cl1-Pd1-P1 = 91.87(5).

The PTA_R3_ ligands were synthesized by the reaction of P(CH_2_NH_2_)_3_ and either benzaldehyde or *p*-*tert*butylbenzylaldehyde under acidic conditions (Scheme 2). X-ray quality crystals of **2** were grown by slow diffusion of diethyl ether into a methylene chloride solution of **2**, Figure 3. Palladium complexes of the PTA_R3_ ligands were prepared by stirring two equivalents of PTA_R3_ and PdCl_2_(COD) in chloroform at room temperature overnight (Scheme 2). The products were obtained as yellow powders in 78% yield for **3** and 83% yield for **4**. The ^31^P-NMR spectrum of **4** contained the expected singlet resonance at −51.4 ppm in CDCl_3_. Compound **3** was obtained as a mixture of *cis* and *trans* isomers as seen by ^31^P-NMR spectroscopy which contained a resonance at −51.5 for the *trans* isomer and one at −34.0 ppm for the *cis* isomer in CDCl_3_. X-ray quality crystals of the *trans* isomers of **3** and **4** were obtained and the structures (Figure 4 and Figure 5) are similar to the previously reported *trans*-PdCl_2_(PTA_PhOMe3_)_2_ [30].

### 2.1. Suzuki-Miyaura Coupling

Initial studies on the catalytic activity of Pd(II) complexes of the water soluble ligands described were performed using phenylboronic acid and bromobenzene in 1:1 H_2_O:CH_3_CN with sodium carbonate as a base and 5% Pd(OAc)_2_ loading. The combination of PTA and Pd(OAc)_2_ produced a modestly active catalyst at 80 °C over 72 h (66%, Table 1, entry 3). No product was observed at room temperature or at 50 °C. The catalyst decomposed quickly to a black precipitate. Formation of black precipitate is not surprising because PTA, with a cone angle of 103°, is not large enough to support the coordinatively unsaturated active catalyst. The addition of mercury to the reactions essentially shut down catalysis (Table 1, entries 4, 5, 7), indicating that colloidal palladium likely was involved.

**Table 1:** Suzuki Coupling catalyzed by Pd(II) and PTA with and without Hg. 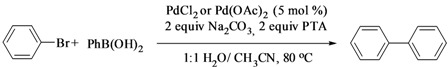

Entry	Catalyst	Time (h)	% Yield ^a^
1	Pd(OAc)_2_PTA_2_	24	36.3
2	Pd(OAc)_2_PTA_2_	48	63.3
3	Pd(OAc)_2_PTA_2_	72	66
4 ^b^	Pd(OAc)_2_PTA_2_	24	3.0
5 ^b^	Pd(OAc)_2_PTA_2_	48	4.9
6	PdCl_2_PTA_2_	48	51.5
7 ^b^	PdCl_2_PTA_2_	48	5.0

^a^ Isolated yield after column chromatography; ^b^ ~0.5 mL Hg added.

PTA derivatives were then employed as ligands in the palladium catalyzed Suzuki coupling (Table 2). All the PTA derivatives explored here resulted in higher yields, 40%–91% than PTA in less time.

**Table 2:** Palladium catalyzed Suzuki coupling reactions in aqueous media utilizing water-soluble PTA derivatives. 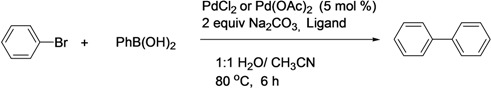

Entry	Precatalyst	Ligand	Pd:L	Yield (%) ^a^
1	Pd(OAc)_2_	DAPTA	1:2	40
2	Pd(OAc)_2_	DAPTA	1:3	42
3	Pd(OAc)_2_	PTA_Ph3_	1:2	65
4	Pd(OAc)_2_	**2**	1:2	56
5	**3**	-	1:2	58
6	**4**	-	1:2	60
7	Pd(OAc)_2_	PTA-CO_2_Li	1:1	60
8	Pd(OAc)_2_	PTA-CO_2_Li	1:2	76
9	Pd(OAc)_2_	PTA-CO_2_Li	1:3	75
10	Pd(OAc)_2_	P(CH_2_NH_3_Br)_3_	1:1	74
11	Pd(OAc)_2_	P(CH_2_NH_3_Br)_3_	1:2	77
12	Pd(OAc)_2_	P(CH_2_NH_3_Br)_3_	1:3	78
13	Pd(OAc)_2_	P(CH_2_NH_2_)_3_	1:1	77
14	Pd(OAc)_2_	P(CH_2_NH_2_)_3_	1:2	80
15	Pd(OAc)_2_	RO-PTA	1:2	86
16	Pd(OAc)_2_	RO-PTA	1:3	82
17 ^b^	Pd(OAc)_2_	RO-PTA	1:2	52
18	PdCl_2_	RO-PTA	1:2	89
19	PdCl_2_	RO-PTA	1:3	85
20	**1**	-	1:1	91
21	Pd(OAc)_2_	-	-	88
22	PdCl_2_	-	-	80

^a^ Isolated yield after column chromatography; ^b^ ~0.5 mL Hg added.

The DAPTA/Pd(OAc)_2_ system provided only slightly better yields, 40%–42%, than PTA (Table 2, entries 1 and 2). Catalysts generated from PTA_R3_/Pd(OAc)_2_ provided the product in moderate yields, 56%–65% (Table 2, entries 3–6). The potentially chelating ligand PTA-CO_2_Li with Pd(OAc)_2_ was slightly more effective with 60%–76% yields depending on the number of equivalents of ligand added (Table 2, entries 7–9). Catalysts derived from P(CH_2_NH_3_Br)_3_ or P(CH_2_NH_2_)_3_ and Pd(OAc)_2_ were more active with yields ranging from 74%–80% (Table 2, entries 10–14). The most active system studied was RO-PTA with Pd(II) salts. The *in situ* catalyst (RO-PTA/Pd(II)) showed very good activity with yields between 82% to 89% depending on ration of ligand to Pd(II) (Table 2, entry 15,16,18,19). The preformed catalyst (**1**) was the most active with yields of 91% obtained for the Suzuki coupling of bromobenzene and phenylboronic acid (Table 2, entry 20).

The ratio of ligand to palladium also affected the amount of product produced. The largest change in yield was observed with PTA-CO_2_Li, as the L:Pd ratio increased from 1:1 to 2:1 the yield increased from 60 to 76%. Increasing the ratio to 3:1 ligand to palladium had little effect on catalysis (Table 2, entry 7–9). Increasing the L:Pd ratio for P(CH_2_NH_3_Br)_3_ (Table 2, entry 10–12) and P(CH_2_NH_2_)_3_ (Table 2, entry 13,14) from 1:1 to 2:1 resulted in only a small increased yield. Increasing the ratio of RO-PTA:Pd(II) from 1:2 to 1:3 resulted in a slight decrease in the coupling product (Table 2, entry 15,16,18,19). Changing the palladium source from Pd(OAc)_2_ to PdCl_2_ with RO-PTA resulted in a small difference in yield with PdCl_2_ being slightly more active (Table 2, entry 15,16,18,19). It is important to note that the reaction remained clear during catalysis for entries 7–16 and 18–19; unlike the PTA/Pd(OAc)_2_ system where palladium black was clearly visible. Addition of Hg to the reaction catalyzed by RO-PTA and Pd(OAc)_2_ resulted in a decrease in yield from 86 to 52% (Table 2, entry 17) indicating that the reaction is mainly homogeneous. RO-PTA and P(CH_2_NH_2_)_3_ ligand are potential (P, N) bidentate ligands and due to the hemilabile functionality [54] the catalyst can be stabilized, reducing the amount of palladium black formed. Ligand free coupling reactions with Pd(OAc)_2_ and PdCl_2_ were explored under the above conditions as a control (Table 2, entry 21–22). Not surprisingly under aqueous conditions Pd(OAc)_2_ and PdCl_2_ are very good catalysts with biphenyl yields of 88 and 80% respectively. This was not unexpected as palladium catalysts have been reported to be excellent heterogenous Suzuki coupling catalysts in aqueous media [4,24,55].

*2.2 Catalyst Scope*

Pd(OAc)_2_, RO-PTA, and sodium carbonate were used to study the catalyst scope under optimized reaction conditions. It is well-established that electron-deficient aryl bromides are good substrates in palladium catalyzed cross coupling reactions. With optimal reaction conditions in hand, the scope of the catalyst system was explored with a range of aryl bromides. Suzuki coupling yields were affected by the steric and electronic parameters of the aryl halides (Table 3). Electron neutral aryl bromides such as 4-bromotoluene (Table 3, entry 1) and electron deficient aryl bromides such as 4-bromo-benzonitrile (Table 3, entry 5) coupled well under the conditions described above. Electron donating aryl bromides such as 1-bromo-4-methoxybenzene (Table 3, entry 4) and sterically demanding aryl bromides such as 2-bromotoluene (Table 3, entry 2) resulted in decreased coupling. No catalytic turnover was observed with the sterically demanding 2-bromo-*m*-xylene (Table 3, entry 3). The sterically demanding election donating 2-bromoanisole resulted in a modest yield (Table 3, entry 6) comparable with electron donating aryl bromide 1-bromo-4-methoxybenzene (Table 3, entry 4). The sterically demanding electron withdrawing 1-bromo-2-nitrobenzene resulted in good but lower yield (Table 3, entry 7) than the electron withdrawing sterically unhindered 4-bromobenzonitrile (Table 3, entry 5). The catalytic activity of RO-PTA/Pd(OAc)_2_ is comparable to Suzuki coupling utilizing water soluble phosphines such as TPPMS and TPPTS [19,20,21]. When compared to water soluble TXPTS and palladacylces developed by Shaughnessy *et al.* [22,23,27] or water soluble diamine ligands [25,26] catalysis by RO-PTA was much less effective.

**Table 3:** Coupling of aryl halides with phenylboronic acid catalyzed by RO-PTA/Pd(OAc)_2_. 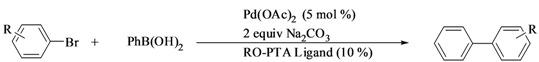

Entry ^b^	Bromide	Precatalyst	Yield (%) ^a^
1	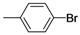	Pd(OAc)_2_	84
2	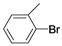	Pd(OAc)_2_	65
3	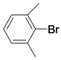	Pd(OAc)_2_	<5
4	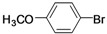	Pd(OAc)_2_	50
5	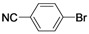	Pd(OAc)_2_	90
6	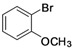	Pd(OAc)_2_	55
7	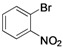	Pd(OAc)_2_	77

^a^ Isolated yield after purification by column chromatography; ^b^ Reactions were run in 1:1 water: acetonitrile at 80 °C for 6 h

## 3. Experimental

### 3.1. General

Standard Schlenk and drybox techniques were used for all reactions unless noted. Prior to use, solvents were distilled under nitrogen from the appropriate drying agent (sodium/benzophenone for tetrahydrofuran, calcium hydride for hexanes; magnesium/iodine for methanol). Water (deionized) and acetonitrile were deoxygented by sparging with nitrogen. Deuterated NMR solvents were purchased from commercial sources and used as received. All NMR spectra were recorded on either a Varian NMR System 400 or Varian Unity Plus 500 FT-NMR spectrometer. ^1^H- and ^13^C-NMR spectra were referenced to a residual solvent relative to tetramethylsilane. Phosphorus chemical shifts are relative to an external reference of 85% phosphoric acid in D_2_O with positive values downfield of the reference. Tetrakis(hydroxymethyl)phosphonium chloride was obtained from Cytec and used without further purification. PTA [44,56], PTA_Ph3_ [30], P(CH_2_NH_3_Br)_3_ [38], P(CH_2_NH_2_)_3_ [30], [Me-PTA]^+^I^−^ [35], DAPTA [36,37], PTA-CO_2_Li [33], and PdCl_2_(COD) [57] were synthesized according to previously reported methods. The synthesis of ROPTA was performed by a modification of a method reported by Schmidbaur [35]. Palladium chloride and palladium acetate were purchased from Strem and stored in a drybox. Aryl bromides and phenylboronic acids were purchased from Acros Organics and used without further purification.

*3,7-Dimethyl-1,5,7-triaza-3-phosphabicyclo[3.3.1]nonane (RO-PTA)*: Prepared by a modification of a method reported by Schmidbaur [35]. To a mixture of condensed liquid ammonia (80 mL) and [Me-PTA]^+^I^−^ (9.00 g, 30.2 mmol) was added sodium metal (878 mg, 38.2 mmol) at −78 °C until the color turned dark blue. Stirring was continued for 20 min at −78 °C. The ammonia was slowly evaporated at room temperature. To the residue was added hexanes (200 mL) and the resulting mixture vigorously stirred for several minutes before filtering under nitrogen. The hexane was removed under reduced pressure resulting in a white, crystalline solid (1.50 g, 29%). Spectral data were identical to previously reported data [35].

*cis-(3,7-Dimethyl-1,5,7-triaza-3-phosphabicyclo[3.3.1]nonane)dichloro palladium (II)*** (1)**: To a solution of PdCl_2_(COD) (142.8 mg, 0.5 mmol) in methylene chloride (15 mL) was added RO-PTA (86.5 mg, 0.5 mmol). Precipitation was observed after 10 min, but the reaction was stirred for another hour. The precipitate was filtered off, washed with methylene chloride (2 × 10 mL), collected and dried *in vacuo* to give the product as a yellow solid (136 mg, 78%). ^1^H-NMR (D_2_O, 400 MHz): δ 4.71 (d, *J* = 12.0 Hz, 2H), 4.18–4.13 (m, 4H),4.02 (d, *J* =12.8 Hz, 2H), 3.86 (d, *J* = 6.8 Hz, 1H), 3.83 (d, *J* = 64 Hz, 1H), 2.52 (s, 3H), 1.67 (d, *J* = 14.4 Hz); ^31^P-NMR (D_2_O, 161.9 MHz): δ −26.6. X-ray quality crystals were obtained by slow evaporation of the dilute solution of chloroform and ether.

*2,4,6-tri(p-tert-Butylphenyl)-1,3,5-phosphatricyclo[3.3.1.1]decane* **(2)**: Tris(aminomethyl)phosphine trihydrobromide (1.091 g, 3.0 mmol) and sodium hydroxide (360 mg, 9 mmol) were added to a 100 mL Schlenk flask in a drybox. Fresh distilled methanol (40 mL) was added via syringe resulting in a clear solution. Hydrogen chloride (30 µL 2.0 M in Et_2_O, 0.06 mmol) and 4-*tert*-butylbenzaldehyde (2.60 mL, 15 mmol) were added to the resulting solution via syringe. The resulting solution was stirred overnight at room temperature. Methanol was removed under reduced pressure. The resultant white residue was dissolved in methylene chloride (100 mL) and sodium bromide was extracted by water (2 × 50 mL). The organic layer was dried over anhydrous potassium carbonate, filtered, and the methylene chloride removed under reduced pressure. The residue was dissolved in methylene chloride (5 mL), absolute ethanol (120 mL) was added, and the flask was set in the freezer overnight. The precipitate was filtered off, washed with ethanol (2 × 15 mL) and dried *in vacuo* to give the product as a white, crystalline solid (1.063, 64%). ^1^H-NMR (CDCl_3_, 400 MHz): δ 7.66–7.40 (m, 6H), 7.51–7.48 (m, 4H), 7.31 (d, *J* = 8.40 Hz, 2H), 5.95 (s, 1H), 5.49 (s, 2H), 4.48 (d, *J*_PH_ = 9.20 Hz, 1H), 4.44 (d, *J*_PH_ = 9.20 Hz, 1H), 3.87 (*J*_PH_ = 9.60 Hz, 1H), 3.84 (d, *J*_PH_ = 10.00 Hz, 1H), 3.73 (d, *J*_PH_ = 9.20 Hz, 2H), 1.37 (s, 18 H), 1.28 (s, 9H). ^13^C-NMR (100.5 MHz, CDCl_3_): δ 150.4, 150.2, 137.3, 136.1, 126.8, 126.1, 125.8, 125.7, 82.9 (d, ^3^*J*_PC_ = 2.90 Hz), 75.1 (d, ^3^*J*_PC_ = 2.70 Hz), 46.4 (d, J = 19.50 Hz), 35.71 (d, *J* = 18.69 Hz), 34.76, 34.6, 31.7, 31.6; ^31^P-NMR (161.7 MHz, CDCl_3_): −111.9. X-ray-quality crystals were obtained by slow diffusion of ether into a methylene chloride solution of ligand **2**, resulting in the formation of clear and colorless blocks over the course of ten days.

*bis(2,4,6-Triphenyl-1,3,5-triaza-7-phosphatricyclo[3.3.1.1]decane)dichloro palladium (II)*** (3)**: To a solution of PdCl_2_(COD) (57.1 mg, 0.2 mmol) in chloroform (15 mL), 2,4,6-triphenyl-1,3,5-triaza-7-phosphatricyclo[3.3.1.1]decane (155.2 mg, 0.4 mmol) in chloroform (5 mL) was added via syringe. The resulting solution was stirred overnight at room temperature and the solvent removed under vacuum. The residue was taken up into a minimum amount of methylene chloride (2 mL), hexanes (20 mL) was added, and the flask was set in the freezer overnight. The precipitate was filtered off, washed with hexanes (2 × 5 mL), and dried under vacuum to give the product as a yellow solid (148 mg, 78%). ^1^H and ^31^P-NMR spectra showed both *cis* and *trans* isomers in CDCl_3_ in a ratio of approximately 1:1. ^1^H-NMR (CDCl_3_, 400 MHz): δ 7.66–7.26 (m, 30 H), 5.99 (s, 2H), 5.95 (s, 2H), 5.45 (s, 1H), 5.43 (s, 1H), 4.52 (s, 2H), 4.48 (s, 2H), 4.16 (s, 2H), 4.12 (s, 2H), 4.02 (s, 2H), 2.94 (s, 2H); ^31^P-NMR (161.7 MHz, CDCl_3_): δ −34.0, −51.5. X-ray-quality crystals were obtained by slow diffusion of ether into a methylene chloride solution of compound **3**.

*trans-bis(2,4,6-tri(p-tert-butylphenyl)-1,3,5-phosphatricyclo[3.3.1.1]decane)dichloro palladium (II)*** (4)**: To a solution of PdCl_2_(COD) (65.6 mg, 0.23 mmol) in chloroform (15 mL) ligand **2** (255.8 mg, 0.46 mmol) in chloroform (5 mL) was added via syringe. The resulting solution was stirred overnight at room temperature, and the solvent removed under vacuum. The residue was taken up into a minimum amount of methylene chloride (2 mL), hexanes (20 mL) was added, and the flask was set in the freezer overnight. The precipitate was filtered off, washed with hexanes (2 × 5 mL), and dried under vacuum to give the product as a yellow solid (256 mg, 83%). ^1^H-NMR (CDCl_3_, 400 MHz): δ 7.54–7.49 (m, 20 H), 7.31–7.31 (m, 4H), 5.97 (s, 2H), 5.40 (s, 4H), 4.52 (s, 2H), 4.48 (s, 2H), 4.22 (s, 2H), 4.18 (s, 2H), 3.97 (s, 4H), 1.37 (s, 36 H), 1.24 (18H) (161.7 MHz, CDCl_3_): δ −51.4. X-ray-quality crystals were obtained by slow diffusion of ether into a methylene chloride solution of compound **4**.

### 3.2. General Procedure for the Suzuki Coupling Reaction Aryl Halides and Arylboronic Acids

A round bottom flask equipped with stir bar in the drybox was charged with palladium chloride or palladium acetate (11.2 mg, 0.05 mmol), an appropriate amount of ligand, sodium carbonate (212 mg, 2.0 mmol) and phenylboronic acid (183 mg, 1.5 mmol). Deoxygenated 1:1 H_2_O:CH_3_CN (5 mL) and aryl halide (1.0 mmol) were added and the reaction was stirred at 80 °C for 6 h unless noted. The reaction was cooled to room temperature, saturated sodium bicarbonate (20 mL) was added, and the organics were extracted with ethyl acetate (3 × 30 mL). The combined ethyl acetate extracts were dried (MgSO_4_) and the solvent was removed under reduced pressure. The crude material was flash chromatographed on a short silica gel column.

### 3.3. Mercury Experiment

A Schlenk flask with stir bar was charged with palladium chloride or palladium acetate (8.8 mg or 11.2 mg respectively, 0.05 mmol), PTA (17.3 mg, 0.10 mmol), and sodium carbonate (212.0 mg, 2.0 mmol) under N_2_ atmosphere. Five mL of deoxygenated H_2_O was then added via syringe and the catalyst solution was stirred for 1.5 h. A 25 mL round bottom was charged with phenylboronic acid (183 mg, 1.5 mmol) and bromobenzene (105 mL, 1.00 mmol), equipped with a condenser under N_2_ atmosphere. Deoxygenated CH_3_CN (5 mL) and catalyst solution (5 mL) were added via syringe. The reaction mixture was stirred for 15 min followed by the addition of a few drops of mercury before heating to 80 °C. After 24 or 48 h the reaction was allowed to cool to room temperature. The mixture was then extracted with dichloromethane (3 × 5 mL). The combined organic extracts were dried over Na_2_SO_4_ and the solvent removed under reduced pressure. The crude product was purified on a short column of silica gel.

*Biphenyl* (Table 2 and Table 3). Bromobenzene (105 μL, 1.00 mmol) and phenylboronic acid (183 mg, 1.5 mmol) were coupled by the above procedure. The product was isolated by column chromatography on silica gel with hexanes as the elution solvent. A white solid was obtained (132 mg, 86%) with spectral data identical to previously reported [58].

*4-Methyl-1,1’-biphenyl* (Table 4, entry 1). 4-Bromotoluene (123 μL, 1.00 mmol) and phenylboronic acid (183 mg, 1.5 mmol) were coupled by the above procedure. The product was isolated by column chromatography on silica gel with hexanes as the elution solvent. A white solid was obtained (133 mg, 84%) with spectral data identical to previously reported [58].

*2-Methyl-1,1’-biphenyl* (Table 4, entry 2). 2-Bromotoluene (120 μL, 1.0 mmol) and phenylboronic acid (183 mg, 1.5 mmol) were coupled by the above procedure. The product was isolated by column chromatography on silica gel with hexanes as the elution solvent. A white solid was obtained (109 mg, 65%) with spectral data identical to previously reported [59].

*4-Methoxy-1,1’-biphenyl* (Table 4, entry 4). 4-Bromoanisole (125 μL, 1.00 mmol) and phenylboronic acid (183 mg, 1.50 mmol) were coupled by the above procedure. The product was isolated by column chromatography on silica gel with 5% ethyl acetate-hexanes as the elution solvent. A colorless oil was obtained (92 mg, 50%) with spectral data identical to previously reported [58].

*4-Cyano-1,1’-biphenyl* (Table 4, entry 5). 4-Bromobenzonitrile (182.0 mg, 1.00 mmol) and phenylboronic acid (183 mg, 1.50 mmol) were coupled by the above procedure. The product was isolated by column chromatography on silica gel with 75:25:5 hexanes-CH_2_Cl_2_-ethyl acetate as the elution solvent. A pale yellow solid was obtained (160.9 mg, 90%) with spectral data identical to previously reported [58].

*2-Methoxy-1,1’-biphenyl* (Table 4, entry 6). 2-Bromoanisole (123.7 μL, 1.00 mmol) and phenylboronic acid (183 mg, 1.50 mmol) were coupled by the above procedure. The product was isolated by column chromatography on silica gel with hexanes as the eluting solvent. A pale yellow oil was obtained (101.3 mg, 55%) with spectral data identical to previously reported [60].

*2-Nitro-1,1’-biphenyl* (Table 4, entry 7). 1-Bromo-2-nitrobenzene (202 mg, 1.00 mmol) and phenylboronic acid (183 mg, 1.50 mmol) were coupled by the above procedure. The product was isolated by column chromatography on silica gel with hexanes and 5% ethyl acetate/hexanes as the elution solvent. A light yellow oil was obtained (153.7 mg, 77%) with spectral data identical to previously reported [61].

### 3.4. X-ray Crystallography

X-ray crystallographic data were obtained on a Bruker APEX CCD diffractometer. The structures were solved by direct methods and refined using SHELXTL, version 6.10 [62,63]. Crystallographic data and data collection parameters may be found in Table 4. CCDC 827744–827747 contains the supplementary crystallographic data for this paper. These data can be obtained free of charge via www.ccdc.cam.ac.uk/conts/retrieving.html.

**Table 4:** Crystallographic data for compounds **1–4**.

	1	2	3	4
Empirical Formula	C_7_H_16_Cl_2_N_3_PPd	C_36_H_48_N_3_P	C_52_H_58_C_l2_N_6_OP_2_Pd	C_74_H_98_C_l8_N_6_P_2_Pd
Fw	350.50	553.74	1022.28	1523.52
T(K)	100(2)	100(2)	100(2)	100(2)
λ(Ǻ)	0.71073	0.71073	0.71073	0.71073
cryst syst	Monoclinic	Triclinic	Triclinic	Triclinic
space group	P21/c	P-1	P-1	P-1
a(Ǻ)	8.60830(10	8.2876(8)	11.10810(10)	12.0121(3)
b(Ǻ)	11.5593(2)	11.8367(12)	14.6003(2)	12.7019(3)
c(Ǻ)	12.0933(2)	17.4678(19)	16.9813(2)	13.9564(3)
α(deg)	90	97.424(7)	113.3050(10)	116.1830(10)
β(deg)	107.1980(10)	100.306(7)	102.5090(10)	94.1170(10)
γ(deg)	90	104.442(8)	100.2660(10)	99.6960(10)
V(Ǻ^3^)	1149.55(3)	1605.2(3)	2359.92(5)	1857.75(8)
Z	4	2	2	1
Dcalc (Mg/m^3^)	2.025	1.146	1.439	1.362
abs coeff (mm^−1^)	2.184	0.114	0.620	0.626
cryst size (mm^3^)	0.07 × 0.06 × 0.03	0.56 × 0.56 × 0.04	0.12 × 0.08 × 0.07	0.31 × 0.09 × 0.05
θ data collect (deg)	2.48 to 26.37	1.81 to 22.50	1.38 to 27.43	1.65 to 27.55
Index ranges	−10 ≤ h ≤ 9	−8 ≤ h ≤ 8	−14 ≤ h ≤ 14	−15 ≤ h ≤ 15
	−14 ≤ k ≤13	−12 ≤ k ≤ 12	−18 ≤ k ≤ 18	−16 ≤ k ≤ 16
	−14 ≤ l ≤ 15	−18 ≤ l ≤ 18	−21 ≤ l ≤ 21	−18 ≤ l ≤ 18
reflns collected	21262	10984	59870	30379
indep reflns	2351 R_int_ = 0.0452	4121 R_int_ = 0.0822	10747 R_int_ = 0.0622	8530 R_int_ = 0.0674
abs correction	SADABS	SADABS	SADABS	SADABS
data/restraints/param	2351/0/127	4121/0/418	10747/0/582	8530/0/421
GOF F^2^	1.088	0.930	1.033	1.051
final R indices	R1 = 0.0343	R1 = 0.0643,	R1 = 0.0424	R1 = 0.0810
[ *I* □ 2σ(*I*)]	wR2 = 0.0761	wR2 = 0.1190	wR2 = 0.0898	wR2 = 0.2168
R indicies	R1 = 0.0462	R1 = 0.1289	R1 = 0.0755	R1 = 0.1200
(all data)	wR2 = 0.0791	wR2 = 0.1363	wR2 = 0.1046	wR2 = 0.2426
CCDC no.	827747	827746	827745	827744

## 4. Conclusions

We have reported here the synthesis and structure of palladium(II) complexes of RO-PTA (**1**) and PTA_R3_ (**3–4**). The air stable, water-soluble, and potentially hemilabile P,N ligand RO-PTA was successfully used for the Suzuki reaction in aqueous media. The combination of RO-PTA and palladium acetate generated an effective catalyst for the Suzuki coupling reaction. Electron neutral and electron deficient aryl bromide substrates coupled well with phenylboronic acid in good yields. The catalytic system was modestly effective in the Suzuki coupling reaction for electron-rich and sterically bulky aryl bromides. Catalytic activity of RO-PTA is comparable to TPPMS and TPPTS and less active than water-soluble diamines and phosphines like TXPTS.
